# Clinical characteristics of elderly patients with proton pump inhibitor-refractory non-erosive reflux disease from the G-PRIDE study who responded to rikkunshito

**DOI:** 10.1186/1471-230X-14-116

**Published:** 2014-07-02

**Authors:** Yasuhisa Sakata, Kazunari Tominaga, Mototsugu Kato, Hiroshi Takeda, Yasuyuki Shimoyama, Toshihisa Takeuchi, Ryuichi Iwakiri, Kenji Furuta, Kouichi Sakurai, Takeo Odaka, Hiroaki Kusunoki, Akihito Nagahara, Katsuhiko Iwakiri, Takahisa Furuta, Kazunari Murakami, Hiroto Miwa, Yoshikazu Kinoshita, Ken Haruma, Shin’ichi Takahashi, Sumio Watanabe, Kazuhide Higuchi, Kazuma Fujimoto, Motoyasu Kusano, Tetsuo Arakawa

**Affiliations:** 1 Department of Internal Medicine and Gastroenterology, Saga Medical School, 5-1-1 Nabeshima, Saga 849-8501, Japan; 2 Department of Gastroenterology, Osaka City University Graduate School of Medicine, Osaka City, Japan; 3 Division of Endoscopy, Hokkaido University Hospital, Hokkaido, Japan; 4 Department of Pathophysiology and Therapeutics, Hokkaido University Faculty of Pharmaceutical Sciences, Hokkaido, Japan; 5 Department of Endoscopy and Endoscopic Surgery, Gunma University Hospital, Gunma, Japan; 6 Second Department of Internal Medicine, Osaka Medical College, Osaka, Japan; 7 Department of Internal Medicine and Gastrointestinal Endoscopy, Saga Medical School, Saga, Japan; 8 Department of Gastroenterology and Hepatology, Shimane University School of Medicine, Shimane, Japan; 9 Department of Gastroenterology, Kumamoto University Graduate School of Medicine, Kumamoto, Japan; 10 Department of Gastroenterology, Chiba University Graduate School of Medicine, Chiba, Japan; 11 Department of Gastroenterology, Kawasaki Medical School, Kawasaki, Japan; 12 Department of Gastroenterology, Juntendo University School of Medicine, Juntendo, Japan; 13 Department of Internal Medicine, Nippon Medical School Chiba Hokusoh Hospital, Nippon, Japan; 14 Center for Clinical Research, Hamamatsu University School of Medicine, Hamamatsu, Japan; 15 Department of Gastroenterology, Faculty of Medicine, Oita University, Oita, Japan; 16 Division of Upper Gastroenterology, Department of Internal Medicine, Hyogo College of Medicine, Hyogo, Japan; 17 Third Department of Internal Medicine, Kyorin Medical College, Kyorin, Japan

**Keywords:** Gastroesophageal reflux, Acid-related dysmotility, Postprandial, Herbal medicine, Elderly patient

## Abstract

**Background:**

The incidence and severity of gastroesophageal reflux disease (GERD) in Japan tends to increase in elderly women. Rikkunshito (RKT), a traditional Japanese medicine, acts as a prokinetic agent and improves gastric emptying and gastric accommodation. Our previous prospective randomized placebo-controlled study showed that RKT combined with a standard-dose of rabeprazole (RPZ) significantly improved the acid-related dysmotility symptoms (ARD) in elderly patients with proton pump inhibitor (PPI)-refractory non-erosive reflux disease (NERD). This study aimed to evaluate clinical characteristics of elderly PPI-refractory NERD patients with ARD symptoms who responded to RKT.

**Methods:**

Two hundred forty-two patients with PPI-refractory NERD were randomly assigned to 8 weeks of either RPZ (10 mg/q.d.) + RKT (7.5 g/t.i.d.) (RKT group) or RPZ + placebo (PL group). Among them, 95 were elderly (≥65 years) with ARD (RKT group: *n* = 52; PL group: *n* = 43). We analyzed the changes using the 12 subscale score of frequency scale for the symptoms of GERD (FSSG) and 15 items of the Gastrointestinal Symptom Rating Scale at 4 and 8 weeks and compared the therapeutic efficacy between the 2 groups.

**Results:**

There were no marked differences in baseline demographic or clinical characteristics in the 2 groups except for rate of current smoking. The FSSG score (mean ± SD at 0, 4, and 8 weeks) in both the RKT (16.0 ± 7.0; 9.9 ± 8.4; 7.0 ± 6.4) and PL (15.1 ± 6.4; 10.9 ± 6.7, 11.1 ± 8.5) groups significantly decreased after treatment. However, the degree of improvement of total and ARD scores of FSSG after the 8-week treatment was significantly greater in the RKT group than in the PL group. Combination therapy with RKT for 8 weeks showed significant improvement in 3 subscale scores (abdominal bloating, heavy feeling in stomach and sick feeling after meals) of the ARD domain and 1 subscale score (heartburn after meals) of the reflux symptom domain.

**Conclusions:**

RKT may be useful for improving GERD symptoms in elderly PPI-refractory NERD patients with ARD. Thus, RKT was particularly effective for resolving postprandial GERD symptoms (heavy feeling in stomach, sick feeling, and heartburn after meals).

**Trial registration:**

(UMIN000005880)

## Background

Gastroesophageal reflux disease (GERD) is a common disorder caused by the reflux of gastric contents into the esophagus [1]. A major therapeutic strategy for GERD is the inhibition of acid secretion using proton pump inhibitors (PPIs) [2]. However, in clinical practice troublesome GERD symptoms persist in 20%–30% of patients despite daily treatment with a standard PPI dose [3]. In particular, the PPI resistance rate (40%–50%) in patients without erosion of the esophageal mucosa [non-erosive reflux disease (NERD)] was higher than that in patients with reflux esophagitis (RE) [4]. Although GERD occurs frequently in Western countries, recent epidemiological studies suggest that the incidence of the disease is increasing in Asian populations [5,6]. Elderly patients are at risk for more severe complications from GERD, and their relative discomfort from the disease is often less than that from comparable pathology for younger patients [7]. One of the features of GERD in Japan is that its frequency and severity tend to increase in elderly women [8]. Previous studies have suggested that risk factors for esophagitis in elderly Japanese women are lumbar kyphosis, short height, hiatus hernia, and negative *Helicobacter pylori* infection status [9,10]. In addition, typical GERD symptoms were frequently observed in elderly patients with GERD at the typical postprandial times in a day, regardless of the presence of esophageal mucosal breaks [11].

In Japan, the traditional medication rikkunshito (RKT), in the form of extracted granules for ethical use (product number TJ-43; Tsumura & CO., Tokyo, Japan) has been approved for medicinal use by the Japanese Ministry of Health and Welfare and is widely prescribed for patients with upper gastrointestinal (GI) symptoms [12]. RKT acts as a prokinetic agent and improves gastric emptying [13] and gastric accommodation [14]. The GERD 4 study revealed that RKT combined with standard-dose rabeprazole (RPZ) decreased the frequency scale for the symptoms of GERD (FSSG) score in patients with PPI-refractory GERD, similar to the decrease observed with treatment with a double dose of RPZ in a randomized, parallel comparative study [15]. Our previous prospective randomized multicenter placebo-controlled study, the G-PRIDE study, showed that RKT may be useful for improving mental quality of life (QOL) and dyspeptic symptoms in patients with PPI-refractory NERD, particularly for elderly and female patients [16]. The aim of this study was to evaluate clinical characteristics of elderly patients with PPI-refractory NERD who responded to RKT from the G-PRIDE study.

## Methods

### Study design and patients

The G-PRIDE study (UMIN000005880) was a prospective, multi-center, randomized, double-blind, paralleled comparative study that examined the pharmacological effects, efficacy, and safety of drug therapy in patients with PPI-refractory NERD in 55 hospitals in Japan. The design and primary results of the prospective clinical trial have been previously described elsewhere [16]. This report includes prospectively defined subgroup analyses of clinical characteristics of elderly patients with PPI-refractory NERD from the G-PRIDE study who responded to RKT. Briefly, 242 patients with PPI-refractory NERD were enrolled in this study from April 2011 to July 2012. Patients with PPI-refractory NERD were defined as those without endoscopic mucosal breaks and with GERD symptoms (FSSG score ≥8) despite a prior therapy with a standard PPI dose (RPZ: 10 mg/day, omeprazole: 20 mg/day, or lansoprazole: 30 mg/day) for ≥4 weeks. PPI-refractory NERD met the following selection criteria: (1) were >20 years of age; (2) had received standard-dose PPI therapy for ≥4 weeks before the start of this study for the treatment of NERD; (3) had an FSSG score ≥8 after standard-dose PPI therapy for ≥4 weeks; (4) planned to receive RPZ (10 mg/day) treatment for ≥8 weeks; and (5) provided written informed consent regarding study participation. Exclusion criteria were as follows: (1) esophageal mucosal erosion in endoscopy carried out within 6 months before the registration; (2) presence of serious complications (liver, kidney, heart, blood, or metabolic disorders); (3) having undergone resection of the upper digestive tract; (4) confirmed presence of a peptic ulcer (excluding ulcer scar) or malignant tumor of the upper digestive tract; (5) inflammatory bowel disease, irritable bowel syndrome (IBS), esophageal stenosis, or esophageal achalasia; (6) diagnosis of a GI motility disorder by the study investigator; (7) suspected organic hepatic/biliary/pancreatic disorders such as gallstone, hepatitis, and pancreatitis; (8) hemorrhage of the digestive tract, mechanical ileus, or perforation of the digestive tract; (9) taking drugs prohibited for concomitant use (such as anti-ulcer drugs except for rabeprazole, prokinetics, other kampo medicines except for RKT) during the observation period; (10) psychoneurosis; (11) receiving or scheduled to receive an agent that is being developed; (12) lactation, pregnancy, or planned pregnancy during the study or follow-up period; (13) intolerance to oral administration; (14) history of allergy for kampo medicine; and (15) considered ineligible to participate by the chief investigator. Patients were randomized to receive RPZ (10 mg once daily) + RKT (7.5 g/day 3 times) (RKT group) or RPZ (10 mg once daily) + placebo (7.5 g/day 3 times) (PL group) for 8 weeks according to a computer-generated randomization list provided by a statistician from the site management organization (SMO) (Sogo Rinsho Holdings Co., Ltd, Tokyo, Japan). After written informed consent was obtained from the study participants, the patients with PPI-refractory NERD who met the inclusion criteria and did not meet the exclusion criteria were recruited for this study. Patients were randomly assigned to the RKT group [RPZ (10 mg/day) + RKT (7.5 g/t.i.d.) for 8 weeks] or the PL group (RPZ + placebo for 8 weeks). RKT (Tsumura & Co., Tokyo, Japan) was used in the form of a powdered extract obtained by spray drying a hot water extract mixture of the following 8 crude herbs: *Atractylodis lanceae rhizoma* (4.0 g), *Ginseng radix* (4.0 g), *Pinellia tuber* (4.0 g), *Hoelen* (4.0 g), *Zizyphi fructus* (2.0 g), *Aurantii nobilis pericarpium* (2.0 g), *Glycyrrhizae radix* (1.0 g), and *Zingiberis rhizoma* (0.5 g). The fingerprint pattern provided by 3-dimensional high-performance liquid chromatography revealed that RKT contains several low molecular compounds (i.e., hesperidin, liquiritin, liquiritigenin, isoliquilitin, isoliquiritigenin, formononetin, glycycoumarin, glycyrrhizin, atractylodin, atractylodinol, 6-shogaol, and 6-gingerol) [17]. Before and after the 4-week and 8-week treatments, GERD symptoms were evaluated using the FSSG questionnaire and GI-related QOL were evaluated using the Gastrointestinal Symptom Rating Scale (GSRS) questionnaire, similar to what was used in earlier clinical reports [18-20]. The study was performed in accordance with the ethical guidelines for clinical studies and considered the patients’ rights and privacy. The study protocol was approved by the institutional review board of each institution (Additional file 1).

### Questionnaire

The FSSG questionnaire comprised 12 items: 2 domains, the reflux symptom (RS) domain, in which the sums of the respective scores of items 1, 4, 6, 7, 9, 10, and 12 are calculated; and the acid-related dysmotility symptom (ARD) domain, in which the sums of the respective scores of items 2, 3, 5, 8, and 11 are calculated (Table 1). The scores were calculated according to the frequency of the symptoms as follows: never, 0; occasionally, 1; sometimes, 2; often, 3; and always, 4 as previously reported. The total score is the sum of the RS and ARD scores, and total scores ≥8 indicated probable GERD as previously validated [19].

**Table 1:** Twelve questions of the FSSG questionnaire

	**Questions**	**ARD/RS**
1	Do you get heartburn?	RS
2	Does your stomach get bloated?	ARD
3	Does your stomach ever feel heavy after meal?	ARD
4	Do you sometimes subconsciously rub your chest with your hand?	RS
5	Do you ever feel sick after meals?	ARD
6	Do you get heartburn after meals?	RS
7	Do you have an unusual (e.g., burning) sensation in your throat?	RS
8	Do you feel full while eating meals?	ARD
9	Do some things get stuck when you swallow?	RS
10	Do you get bitter liquid (acid) coming up into your throat?	RS
11	Do you burp a lot?	ARD
12	Do you get heartburn if you bend over?	RS

RS, reflux symptom; ARD, acid-related dysmotility symptom.

The GSRS questionnaire is an inquiry table consisting of 15 items for the evaluation of general GI symptoms [20]. Each GSRS item is rated on a 7-point Likert scale from no discomfort to very severe discomfort. Based on a factor analysis, the 15 GSRS items break down into the following 5 scales: abdominal pain (abdominal pain, hunger pain, and nausea), reflux syndrome (heartburn and acid regurgitation), diarrhea syndrome (diarrhea, loose stools, and urgent need for defecation), indigestion syndrome (borborygmus, abdominal distension, eructation, and increased flatus), and constipation syndrome (constipation, hard stools, and a feeling of incomplete evacuation).

### Clinical characteristics of elderly patients with PPI-refractory NERD who responded to RKT

Subgroup analysis was pre-planned to perform with respect to each subject’s background factors such as age (≥65 years or ≤64 years), gender (male or female), body mass index (BMI ≥22 or <22), classification as ARD and RS scores of the FSSG. The patients aged ≥65 years with ARD symptoms were selected from the patients with PPI-refractory NERD. A patient who had one or more ARD symptom score of FSSG was defined as a patient with ARD symptoms. Clinical characteristics of elderly patients with PPI-refractory NERD who responded to RKT were assessed by improvement degrees of 12 subscale scores of FSSG and 15 GSRS items after the treatment.

Improvement degree was calculated based on the FSSG or GSRS score before and after treatment, using the following mentioned formula. To compare the effects between the 2 groups, the mean improvement degree was used: (FSSG and GSRS) improvement degree (Δ) = [pre scores] − [post scores]. In addition, rikkunshito responder was defined as those whom the FSSG score has improved 50% or more after the 8-week treatment of RKT, and the differences in clinical characteristics between the RKT responsive group and nonresponsive group were investigated.

### Statistical analysis

The efficacy analysis was based on the full analysis set (FAS) population. The treatment response in each group was evaluated based on changes in the FSSG and GSRS questionnaire scores before and after treatment, using Wilcoxon’s signed rank test. We employed the *t-*test to compare background factors such as age and BMI. The distributions of gender, current alcohol use, current smoking status, *H. pylori* infection, gastric mucosal atrophy, gastric mucosa redness, impaired gastroesophageal flap valve (GEFV), and esophageal hiatal hernia (grades B and A) were compared using Fisher’s exact test. Values of *P* < 0.05 were considered statistically significant. All data are expressed as mean ± standard deviation (S.D.).

## Results

### Eligible patients

**Figure 1:**
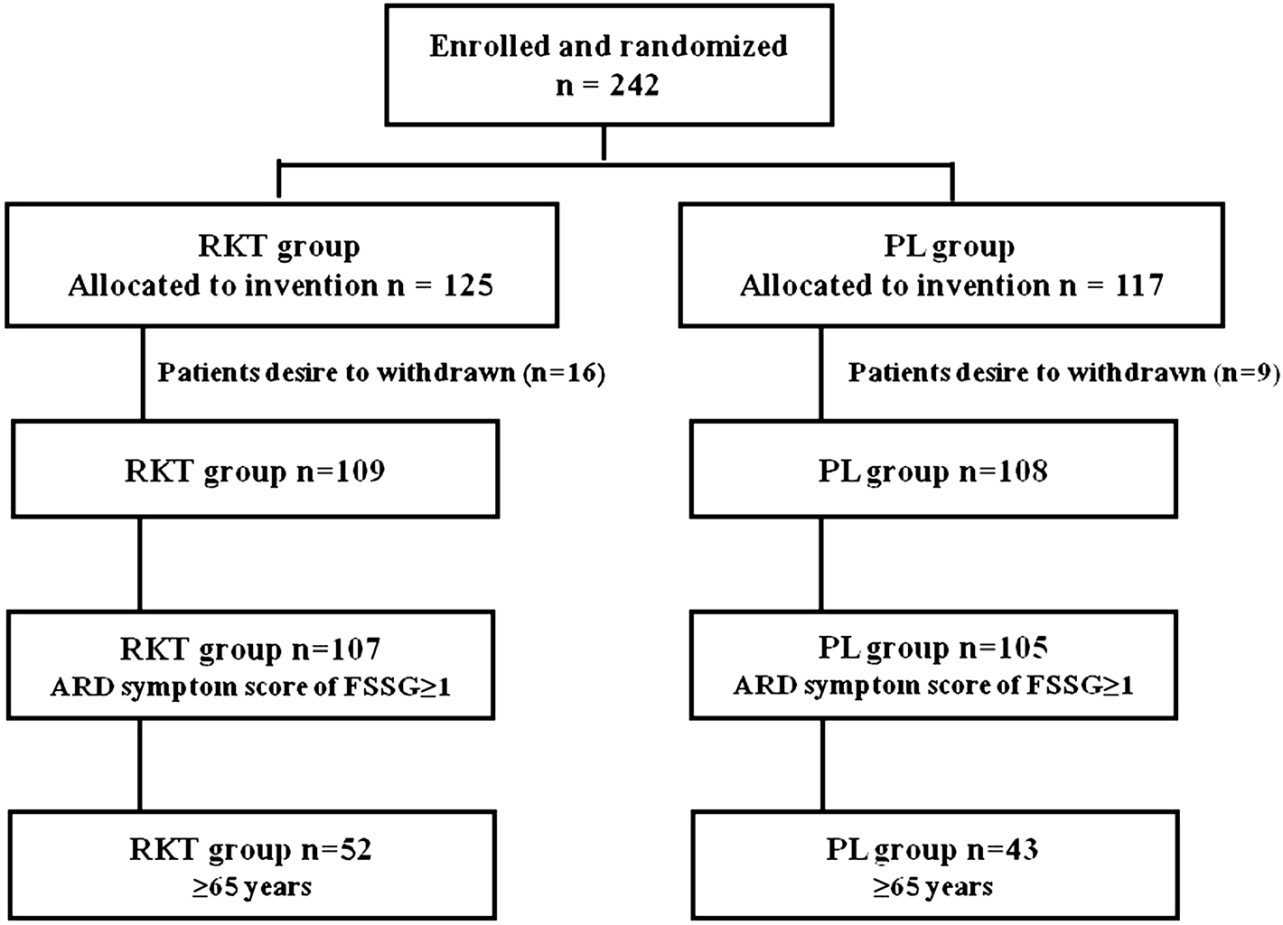
**Patients enrolled in this study.** Of 242 patients with a clinical diagnosis of PPI-refractory NERD, 95 were elderly patients (≥65 years) with ARD. ARD, acid-related dysmotility symptom; RKT group: rikkunshito (7.5 g/day 3 times) + rabeprazole (10 mg/day), PL group: placebo (7.5 g/day 3 times) + rabeprazole (10 mg/day).

Two hundred forty-two patients were randomly assigned to the RKT group (n = 125) or the PL group (n = 117). Twenty-five patients were excluded from the efficacy assessment because they withdrew from the study after registration (RKT group: *n* = 109, PL group: *n* = 108). Of the 217 patients, 95 were elderly patients (≥65 years) with ARD (RKT group: *n* = 52; PL group: *n* = 43) (Figure 1).

### Background of elderly PPI-refractory NERD patients with ARD

There were no marked differences in baseline demographic or clinical characteristics in the 2 groups except for rate of current smoking. Furthermore, there was no difference in total FSSG or overall GSRS scores before the start of treatment between the 2 groups (Table 2).

**Table 2:** Patients background factors in the 2 groups

	**RKT group**	**PL group**	***P *value**
Mean age, years (range)	72.1 (65–85)	73.4 (65–83)	0.225^a^
Gender, *n*, (M/F)	17/35	8/35	0.370^b^
BMI (mean ± SD)	23.0 ± 3.6	22.9 ± 3.0	0.940^a^
Current alcohol use, *n* (%)	7 (13.5)	3 (7.0)	0.517^b^
Current smoking, *n* (%)	6 (11.5)	1 (2.3)	0.024^b^
*Helicobacter pylori* infection, *n* (%)	13 (43.3)	5 (16.7)	0.115^b^
Gastric mucosal atrophy, *n* (%)	37 (71.2)	27 (64.3)	0.096^b^
Redness of gastric mucosa, *n* (%)	10 (19.2)	3 (7.1)	0.167^b^
Impaired GEFV (grade III, IV), *n* (%)	16 (30.8)	9 (20.9)	0.427^b^
Esophageal hiatal hernia (grade B and A), *n* (%)	23 (48.6)	17 (39.7)	0.413^b^
Concomitant systemic diseases, *n* (%), (with)	34 (65.4)	26 (61.9)	0.785^b^
Total FSSG score (mean ± SD)	16.0 ± 7.0	15.1 ± 6.4	0.457^c^
Overall GSRS score (mean ± SD)	2.5 ± 0.9	2.3 ± 0.8	0.652^c^

BMI, body mass index; GEFV, gastroesophageal flap valve; FSSG, Frequency Scale for the Symptoms of GERD; GSRS, Gastrointestinal Symptom Rating Scale.

^a^, *t*-test; ^b^, Fisher’s exact test; ^c^, Wilcoxon’s signed rank test.

### Changes in FSSG scores after treatments

**Figure 2:**
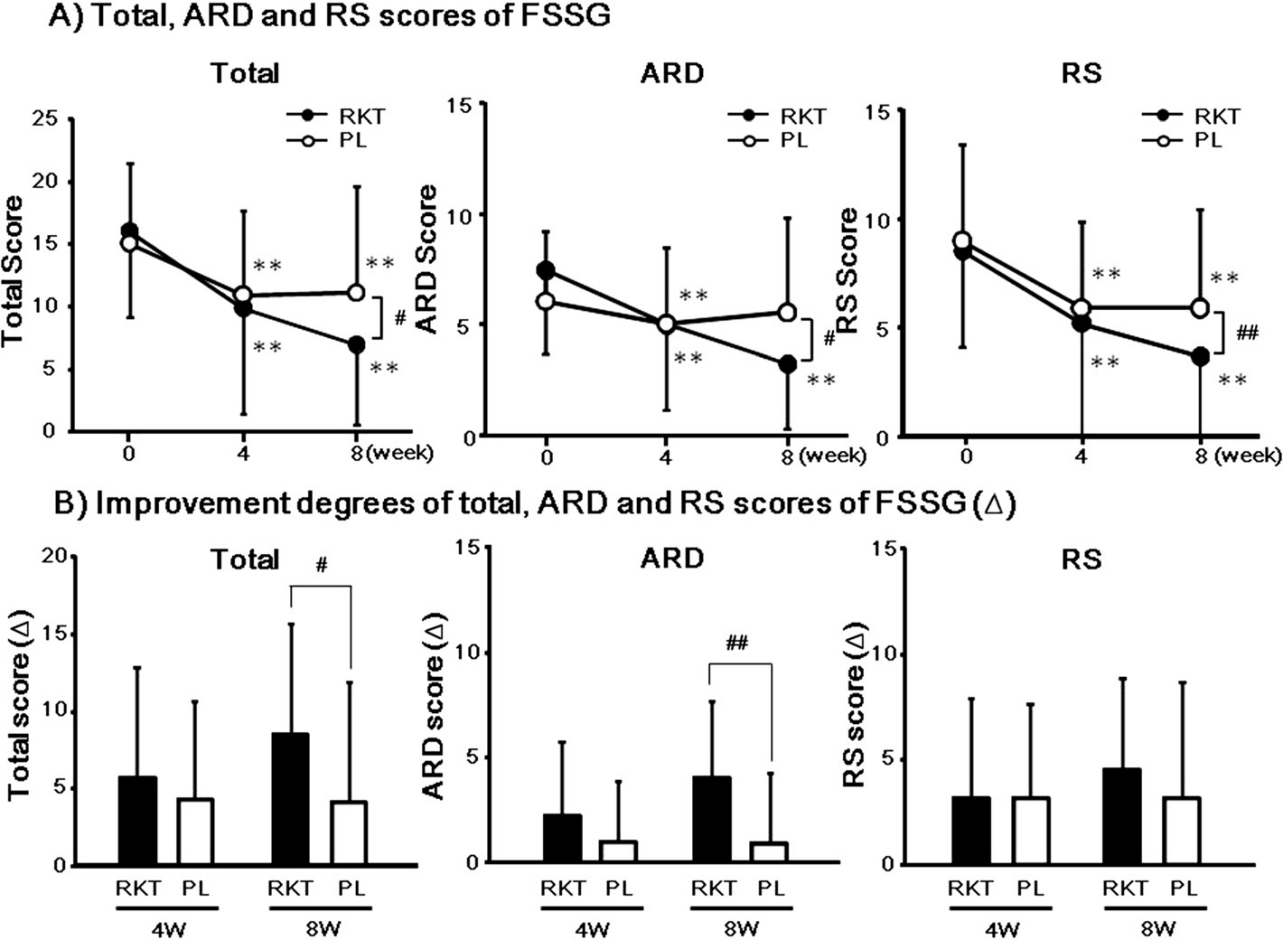
**Changes in FSSG scores after treatments of RKT or placebo. (A)** Changes in total, ARD and RS scores of FSSG after the 4- and 8-week treatment. **(B)** Improvement degrees of total, ARD and RS scores of FSSG after the 4- and 8-week treatment. Values are expressed as mean ± SD. **P < 0.01 vs. before treatment (Wilcoxon’s signed rank test). ^#^*P* < 0.05, ^##^*P* < 0.01 significant difference between each paired group (Wilcoxon’s signed rank test).

Changes in total, ARD, and RS scores of FSSG after treatments in each group are shown in Figure 2A. In both groups, total, ARD, and RS scores of FSSG were significantly decreased after the 4-week treatment compared with that before treatment (*P* < 0.01). Total, ARD, and RS scores of FSSG in the RKT group but not in the PL group further decreased during 4–8 weeks after treatment. Improvement degrees of total, ARD, and RS scores of FSSG after treatments in each group are shown in Figure 2B. The improvement degrees of the total and ARD scores but not RS score of FSSG were significantly higher in the RKT group than in the PL group after the 8-week treatment.

### Improvement degrees of 12 subscale scores of FSSG after the 8-week treatment

**Figure 3:**
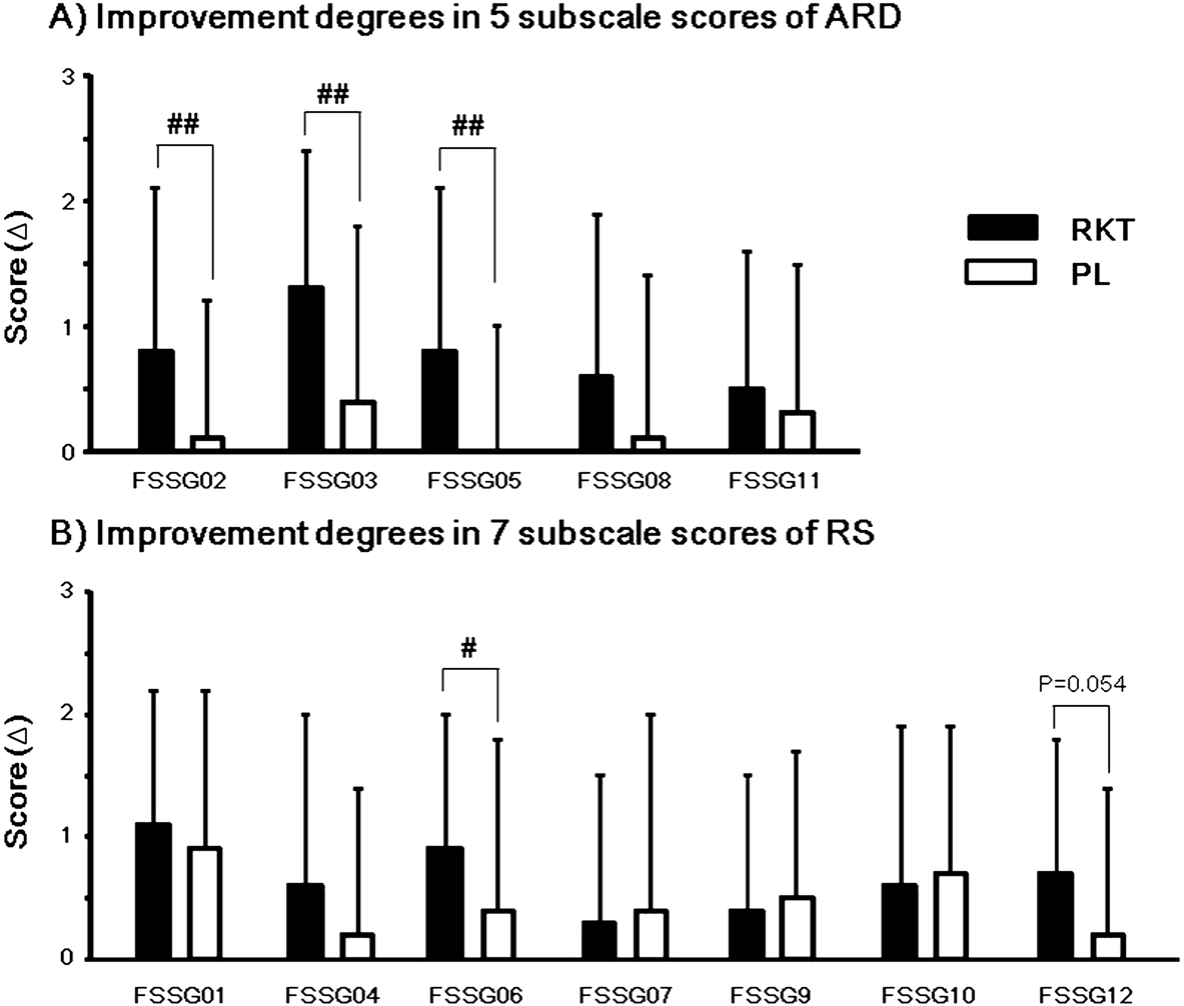
**Improvement degrees of 12 subscale scores of FSSG after the 8-week treatment in the RKT and placebo 2 groups. (A)** Improvement degrees in 5 subscale scores of ARD after the 8-week treatment. **(B)** Improvement degrees in 7 subscale scores of RS after the 8-week treatment. Values were expressed as mean ± SD. ^#^*P* < 0.05, ^##^*P* < 0.01 significant difference between each paired group (Wilcoxon’s signed rank test).

Out of 5 subscale scores of ARD, improvement degrees of FSSG-02 (abdominal bloating), FSSG-03 (heavy feeling in stomach after meals), and FSSG-05 (sick feeling after meals) scores were significantly higher in the RKT group than in the PL group (Figure 3A). Out of 7 subscale scores of RS, improvement degree of FSSG-06 (heartburn after meals) score was significantly higher in the RKT group than in the PL group (Figure 3B). Improvement degree of FSSG-12 (heartburn when bending over) score tended to be higher in the RKT group than in the PL group (*P* = 0.054, Figure 3B).

### Improvement degrees of GSRS scores after the 8-week treatment

Improvement degrees of GSRS scores after the 8-week treatment in each group are presented in Table 3. Improvement degrees of reflex syndrome score of GSRS tended to be higher in the RKT group than in the PL group (*P* = 0.064). However, there was no significant difference in the degree of improvement of the overall and 5 subscale scores of GSRS between the 2 groups after the 8-week treatment. Of the 15 GSRS items, improvement degrees of GSRS-03 (acid regurgitation), GSRS-07 (abdominal distension), and GSRS-10 (constipation) scores were significantly higher in the RKT group than in the PL group.

**Table 3:** Improvement degrees of GSRS after 8-week treatments of RKT or placebo

	**RKT group**	**PL group**	*** P***	**Subscale domain**
(Questions)				
GSRS01 (abdominal pain)	0.5 ± 1.4	0.7 ± 1.7	0.425	Abdominal pain
GSRS02 (heartburn)	1.4 ± 1.4	0.8 ± 1.8	0.240	Reflux syndrome
GSRS03 (acid regurgitation)	1.4 ± 1.9*	0.6 ± 1.6	0.038	Reflux syndrome
GSRS04 (hunger pains)	0.6 ± 1.2	0.6 ± 1.4	0.785	Abdominal pain
GSRS05 (nausea)	0.8 ± 1.6	0.1 ± 1.5	0.062	Abdominal pain
GSRS06 (borborygmus)	0.6 ± 1.4	0.5 ± 1.4	0.944	Indigestion syndrome
GSRS07 (abdominal distension)	1.0 ± 2.0*	0.2 ± 1.8	0.038	Indigestion syndrome
GSRS08 (eructation)	1.0 ± 1.4	0.3 ± 1.9	0.197	Indigestion syndrome
GSRS09 (increased flatus)	0.8 ± 1.4	0.6 ± 1.8	0.524	Indigestion syndrome
GSRS10 (constipation)	0.8 ± 1.6*	0.2 ± 1.4	0.034	Constipation syndrome
GSRS11 (diarrhea)	0.5 ± 1.5	0.6 ± 1.4	0.661	Diarrhea syndrome
GSRS12 (loose stools)	0.3 ± 1.5	0.6 ± 1.5	0.260	Diarrhea syndrome
GSRS13 (hard stools)	0.6 ± 1.5	0.4 ± 1.5	0.479	Constipation syndrome
GSRS14 (urgent need for defecation)	0.6 ± 1.4	0.4 ± 1.5	0.339	Diarrhea syndrome
GSRS15 (feeling of incomplete evacuation)	0.5 ± 1.4	0.4 ± 1.5	0.518	Constipation syndrome
(Subscale score)				
Abdominal pain	0.6 ± 1.0	0.5 ± 1.3	0.538	
Reflux syndrome	1.4 ± 1.5	0.7 ± 1.6	0.064	
Indigestion syndrome	0.8 ± 1.2	0.4 ± 1.4	0.211	
Constipation syndrome	0.6 ± 1.2	0.3 ± 1.3	0.094	
Diarrhea syndrome	0.5 ± 1.3	0.5 ± 1.3	0.677	
Overall score	0.8 ± 0.9	0.5 ± 1.1	0.113	

GSRS, Gastrointestinal Symptom Rating Scale; the 15 GSRS items are divided into the following 5 scales: abdominal pain (abdominal pain, hunger pain, and nausea), reflux syndrome (heartburn and acid regurgitation), diarrhea syndrome (diarrhea, loose stools, and urgent need for defecation), indigestion syndrome (borborygmus, abdominal distension, eructation, and increased flatus), and constipation syndrome (constipation, hard stools, and a feeling of incomplete evacuation). Values were expressed as mean ± SD. *P < 0.05, significant difference between each paired group (Wilcoxon’s signed rank test).

### The differences in clinical characteristics between the RKT responsive group and RKT nonresponsive group

Background factors of RKT responsive group and RKT nonresponsive group were shown in Table 4. Presence of concomitant systemic diseases (hypertension, insomnia, hyperlipidemia and constipation) was the potential predictor of poor response to RKT.

**Table 4:** The differences in clinical characteristics between the RKT responsive group and RKT nonresponsive group

	**Responders (n = 25)**	**Non-responders (n = 17)**	**P value**
Mean age, years (range)	71.12(65-85)	73.12(65-85)	0.441^a^
Gender, *n*, (M/F)	10/15	4/13	0.331^b^
BMI (mean ± SD)	23.5 ± 4.1	22.3 ± 2.3	0.497^a^
Current alcohol use (Y/N)	4/21	1/16	0.632^b^
Current smoking (Y/N)	5/20	0/17	0.070^b^
*Helicobacter pylori* infection (Y/N)	6/8	4/6	1.000^b^
Gastric mucosal atrophy,	18/7	11/6	0.738^b^
Redness of gastric mucosa (Y/N)	5/20	2/15	0.681^b^
GEFV(grade I&II/III&IV)	17/8	10/7	0.744^b^
Esophageal hiatal hernia (grade O&C/B&A)	13/12	9/8	1.000^b^
Concomitant systemic diseases(Y/N))	13/12	15/2	0.020^b^
Total FSSG score (mean ± SD)	15.9 ± 7.4	14.6 ± 6.5	0.616^a^
Total GSRS score (mean ± SD)	2.5 ± 1.0	2.3 ± 0.8	0.722^a^

BMI, body mass index; GEFV, gastroesophageal flap valve; FSSG, Frequency Scale for the Symptoms of GERD; GSRS, Gastrointestinal Symptom Rating Scale.

^a^, Wilcoxon’s rank sum test; ^b^, Fisher’s exact test.

## Discussion

In this population of elderly PPI-refractory NERD patients with ARD, RKT combined with a standard-dose of RPZ significantly improved the total and ARD scores but not RS score of FSSG after an 8-week treatment. Our results suggest that for the improvement of postprandial dyspeptic symptoms in elderly patients with PPI-refractory NERD, RKT is particularly effective. Furthermore, RKT was more effective in patients without concomitant systemic diseases.

Kindt et al. have reported a relationship between symptom pattern and gastric sensorimotor dysfunction in functional dyspepsia [21]. This report suggested that gastric emptying was correlated with scores for heartburn/regurgitation, nausea/vomiting, fullness/satiety, bloating, and lower abdominal pain. However, discomfort volume during gastric distension was correlated with scores for fullness/satiety, bloating, heartburn/regurgitation, and upper abdominal pain. In the present study, clinical characteristics of elderly patients with PPI-refractory NERD who responded to RKT were assessed by improvement degrees of 12 subscale scores of FSSG and 15 GSRS items after the 8-week treatment. Combination therapy with RKT for 8 weeks showed significant improvement in 3 subscale scores (abdominal bloating, heavy feeling in stomach after meals, and sick feeling after meals) of the ARD domain and 1 subscale score (heartburn after meals) of the RS domain. Transient lower esophageal sphincter relaxations (TLESRs) are one of the major mechanisms for reflux in GERD. Delayed gastric emptying and impaired gastric accommodation may trigger TLESRs. RKT acts as a prokinetic agent that ameliorates gastric emptying [13] and gastric accommodation [14]. The increase of gastric motor activity by RKT may contribute to improve postprandial GERD symptoms in elderly patients with PPI-refractory NERD.

Of the 15 GSRS items, improvement degrees of GSRS-10 (constipation) scores were significantly higher in the RKT group than in the PL group. It is known that voluntary suppression of defecation delays gastric emptying in healthy subjects [22]. The improvement that RKT has on constipation may also be because of its prokinetic effect. A previous clinical study of 340 Japanese patients with GERD reported that elderly patients with GERD frequently demonstrated typical GERD symptoms at the typical postprandial time points in a day, regardless of the esophageal mucosal breaks presence [11]. Because RKT was particularly effective for resolving postprandial GERD symptoms in the present study, it may be suitable for treatment of elderly patients with GERD.

Recently, esophageal impedance pH monitoring revealed that bile reflux to the esophagus may be another important factor in the pathogenesis of mucosal hypersensitivity leading to PPI-refractory GERD. Araki et al. showed that RKT can potently absorb bile salts [23]. In addition, RKT improved acid regurgitation-associated signs through prevention of both decreases in the tight junction protein and increases in the intercellular space of the epithelial mucosa in a rat reflux esophagitis model [24]. These findings suggest that RKT may improve hypersensitivity against gastric acid or bile reflux to the esophagus. These mechanisms, which are unrelated to motor function, could explain the effect of RKT on relieving the symptoms of patients with PPI-refractory GERD, particularly in NERD. In addition to these effects, RKT enhances ghrelin secretion and reactivity of its receptor [25-27]. Ghrelin is a digestive hormone, which displays a wide spectrum of biological functions including appetite stimulation, GI motility, and gastric mucosal protection [28,29]. Interestingly, it was reported that RKT improved decreased food intake in elderly patients with dementia [30] and aged mice [31]. Elderly patients with GERD have complications such as dysphagia, vomiting, weight loss, anemia, and anorexia [32]. Therefore, RKT may be a possible treatment to improve QOL in elderly patients with GERD/NERD.

The placebo response in functional GI diseases including GERD/NERD, functional dyspepsia (FD), and irritable bowel syndrome (IBS) is a significant confounder of the assessment of drug efficacy in clinical trials. Though FSSG scores significantly decreased in the PL group during the first 4 weeks of treatment, significant changes of the scores were not observed during 4–8 weeks. In contrast, FSSG scores significantly decreased in RKT group during 4–8 weeks. Therefore, it is believed that the significant difference was observed in the improvement degree between 2 groups only in the 8^th^ week after treatment. When a patient with ≥50% improvement rate of FSSG score is defined as a placebo responder, the placebo response rates in this population of elderly PPI-refractory NERD patients with ARD were 34.2% and 41.9% after 4 or 8 weeks of treatment, respectively (data not shown). Previous reports showed that placebo response rate in GERD patients was approximately 19% [33]. This rate is lower than placebo response rate in FD or IBS patients (approximately 40%) [34-36]. High placebo response rate in our cohort may be related to patients overlapping with FD and/or IBS who unfortunately have been included. We have fully recognized the necessity for physiological testing using multichannel intraluminal impedance-pH monitoring to distinguish NERD patients form FD patients. However, it is difficult to perform such physiological testing in all clinical institutions. This point was an unavoidable limitation in multicenter clinical trials as well as other similar clinical trials

## Conclusions

RKT is a potential medicine in improving GERD symptoms in elderly PPI-refractory NERD patients with ARD. On the basis of our results, we can conclude that RKT was particularly effective for treatment of postprandial GERD symptoms (heavy feeling in stomach after meals, sick feeling after meals, and heartburn after meals).

## Abbreviations

ARD: Acid-related dysmotility symptom; BMI: Body mass index; FAS: Full analysis set; FD: Functional dyspepsia; FSSG: The frequency scale for the symptoms of GERD; GEFV: Gastroesophageal flap valve; GERD: Gastroesophageal reflux disease; GSRS: The Gastrointestinal symptom rating scale; IBS: Irritable bowel syndrome; NERD: Non-erosive reflux disease; QOL: Quality of life; RE: Reflux esophageal; RKT: Rikkunshito; RPZ: Rabeprazole; RS: Reflux symptom; PPI: Proton pump inhibitor; SMO: Site management organization; TLESRs: Transient lower esophageal sphincter relaxations.

## Competing interests

Some authors have received research grants, respectively: Mototsugu Kato from Otsuka Pharmaceutical Co. Ltd., Takeda Pharmaceutical Co., Ltd., AstraZeneca KK., Ltd., Astellas Pharma Inc., and Daiichi-Sankyo Co., Ltd.; Hiroshi Takeda from Tsumura & Co. Ltd., Eiji Umegaki from Takeda Pharmaceutical Co., Ltd.; Akihito Nagahara from Daiichi-Sankyo Co., Ltd.; Katsuhiko Iwakiri from Takeda Pharmaceutical Co.; Yoshikazu Kinoshita has served in speaking and teaching commitments for AstraZeneca KK, Takeda Pharmaceutical Co., Ltd., Astellas Pharma Inc., Eisai Co., Ltd., Taiho Pharmaceutical Co., Ltd., Daiichi-Sankyo, and MSD; ST from Eisai Co., Ltd.; Sumio Watanabe from AstraZeneca Inc., Eisai Co., Ltd., and Takeda Pharmaceutical Co., Ltd.; Kazuhide Higuchi from AstraZeneca KK, Otsuka Pharmaceutical Co. Ltd., Eisai Co., Ltd., Daiichi-Sankyo Co. Ltd., and Nippon-Shinyaku Co. Ltd.; Motoyasu Kusano from Eisai Co., Ltd.; Kazuma Fujimoto from Eisai Co., Ltd., AstraZeneca KK., and Daiichi-Sankyo Co. Ltd.; Tetsuo Arakawa from Eisai Co., Ltd., Daiichi-Sankyo Co. Ltd., and Otsuka Pharmaceutical Co., Ltd. The Center for Clinical Research at Hamamatsu University School of Medicine has received grants from Takeda Pharmaceutical Co., Ltd., AstraZeneca KK, Eisai Co., Ltd., and Daiichi-Sankyo Co. Ltd., and Takahisa Furuta has received lecture fees from those companies. The Division of Upper Gastroenterology, Department of Internal Medicine at Hyogo College of Medicine has received grants from AstraZeneca KK, Astellas Pharma Inc., Otsuka Pharmaceutical Co. Ltd., Eisai Co., Ltd., Tsumura & Co. Ltd., Dainippon Sumitomo Pharma Co. Ltd., Yakult Co. Ltd., and Takeda Pharmaceutical Co., Ltd. The following people have nothing to declare: Yasuhisa Sakata, Kazunari Tominaga, Yasuyuki Shimoyama, Ryuichi Iwakiri, Kenji Furuta, Koichi Sakurai, Takeo Odaka, Hiroaki Kusunoki, Kazunari Murakami, and Ken Haruma.

Financial support for this study was provided by the Waksman Foundation of Japan INC.

## Authors’ contributions

Contributions to study concept and design, interpretation of data, drafting of the manuscript, critical revision of the manuscript for important intellectual content: KT, MK, HT, YS, EU, RI, YK, KH, KH, MK, KF, and TA. Contributions to drafting of the manuscript and critical revision of the manuscript for important intellectual content: YS, KF, KS, TO, HK, AN, and KI. Contributions to critical revision of the manuscript for important intellectual content: TF, KM, and HM. Contributions to study concept and design, interpretation of data, critical revision of the manuscript for important intellectual content: KH, ST, and SW. All authors approved the final version of the manuscript.

## Pre-publication history

The pre-publication history for this paper can be accessed here:

http://www.biomedcentral.com/1471-230X/14/116/prepub

## Supplementary Material

Additional file 1 Lists of the institutional review boards. Click here for file
