# Supplementary material for: Clinical characteristics of elderly patients with proton pump inhibitor-refractory non-erosive reflux disease from the G-PRIDE study who responded to rikkunshito
Source: BMC Gastroenterol. 2014 Jul 2;14:116. doi: 10.1186/1471-230X-14-116 (PMC4090183; doi:10.1186/1471-230X-14-116)
Supplement: Additional file 1 — Lists of the institutional review boards. [file 1471-230X-14-116-S1.docx]

**Lists of the institutional review boards**

1. Hokkaido University Hospital Institutional Review Board
2. Sapporo Hokuyu Hospital Ethical Committee
3. Tomakomai City Hospital Ethical Committee
4. Sapporo Higashi Tokushukai Hospital Institutional Review Board
5. Keiyukai Sapporo Hospital Ethical Committee
6. Nikko Memorial Hospital Ethical Committee
7. Gunma University Hospital Institutional Review Board
8. Takasaki General Medical Center Institutional Review Board
9. Haramachi Red Cross Hospital Ethical Committee
10. Jyuntendo University School of Medicine Institutional Review Board
11. Kyorin University Hospital Ethical Committee
12. Nippon Medical School Hospital Institutional Review Board
13. Chiba University Hospital Institutional Review Board
14. Tako Central Hospital Ethical Committee
15. Hamamatsu University Hospital Ethical Comittee
16. Fujieda Municipal General Hospital Ethical Committee
17. Enshu Hospital Ethical Committee
18. Yaizu City General Hospital Ethical Committee
19. Osaka City University Hospital Ethical Committee
20. Ohno Memorial Hospital Ethical Committee
21. Higashisumiyoshi Morimoto Hospital Ethical Committee
22. Meijibashi Hospital Ethical Committee
23. Nagayoshi General Hospital Ethical Committee
24. Osaka Medical College Hospital Ethical Committee
25. Osaka Saiseikai Nakatsu Hospital Ethical Committee
26. Moriguchi Keijinkai Hospital Ethical Committee
27. Hokusetsu General Hopital Ethical Committee
28. Seikeikai Hospital Ethical Committee
29. Shinsei Hospital Ethical Committee
30. Hyogo College of Medicine Hospital Ethical Committee
31. Takarazuka City Hospital Ethical Committee
32. Kawasaki Medical School Hospital Ethical Committee
33. Shimane University Hospital Ethical Committee
34. Izumo City General Medical Center Ethical Committee
35. Shimane Prefectural Central Hospital Institutional Review Board
36. Matsue Red Cross Hospital Ethical Committee
37. Saga University Hospital Ethical Committee
38. Ureshino Medical Center Ethical Committee
39. Saga Prefectural Hospital Koseikan Ethical Committee
40. Imari Arita Kyoritsu Hospital Institutional Review Board
41. Karatsu Red Cross Hospital Ethical Committee
42. Kumamoto Medical Center Ethical Committee
43. Oita University Hospital Institutional Review Board
44. Nagasaki University Hospital Ethical Committee
45. Central Institutional Review Board of Sogo Rinsho Science
